# Medical-Legal Partnerships: a promising approach for addressing health-harming legal needs among people with HIV

**DOI:** 10.3389/fsoc.2024.1422783

**Published:** 2024-07-09

**Authors:** Julia Jaén, Anne Frankel, Ashley French, Robin Davison, Miguel Munoz-Laboy, Omar Martinez

**Affiliations:** ^1^Social and Behavioral Sciences, Temple University, Philadelphia, PA, United States; ^2^College of Medicine, University of Central Florida, Orlando, FL, United States; ^3^School of Social Work, Stony Brook University, New York, NY, United States

**Keywords:** HIV care, Medical-Legal Partnerships, health-harming legal needs, structural racism and discrimination, social-ecological model

## Abstract

**Introduction:**

People with HIV (PWH), particularly those at the intersection of sexual and gender identities, face enduring obstacles to accessing HIV care, including structural stigma, structural racism and discrimination, housing instability, and limited access to health insurance. To address these challenges, Medical-Legal Partnerships (MLPs) in HIV care offer an innovative approach that integrates medical and legal services. By targeting health-harming legal needs (HHLN), MLPs aim to enhance the HIV care continuum outcomes for PWH.

**Methods:**

This study examines the benefits and challenges of MLPs within organizations serving PWH through the social-ecological model. MLP providers (*n*=111) identified organizational-level challenges such as funding limitations, resource integration issues, and staffing constraints.

**Results:**

MLPs demonstrated numerous benefits, including patient impact and benefits, comprehensive service provision, enhanced staff support and capacity, and potential for policy influence.

**Discussion:**

These results underscore the feasibility of MLPs while offering valuable insights into their efficacy and challenges, guiding the implementation of MLPs to address health-harming legal needs, including discrimination, and thereby improving HIV care outcomes.

## Introduction

People with HIV (PWH) encounter myriad obstacles that impede their access to essential HIV care across various societal domains (Kinsky et al., 2015; Guilamo-Ramos et al., 2020; Miller et al., 2021; Erickson et al., 2022). Structural stigma significantly impacts people living with HIV and their access to HIV care by perpetuating discriminatory policies and social norms that hinder their ability to seek and receive adequate healthcare services and support (Wiginton et al., 2023; Fisk-Hoffman et al., 2024). Structural racism and discrimination perpetuate systemic inequalities within healthcare systems, disproportionately affecting marginalized communities, including racial and ethnic minorities (Arevalo, 2018). These discriminatory practices not only create barriers to accessing quality care but also contribute to disparities in health outcomes among PWH. Moreover, housing instability poses a significant challenge for individuals living with HIV, as unstable housing situations can disrupt continuity of care, medication adherence, and overall health management (Aidala et al., 2016; Fernandez et al., 2022). Additionally, limited access to health insurance further exacerbates the difficulties faced by PWH, as it impedes their ability to afford necessary medical services and medications, resulting in suboptimal health outcomes (Bachman et al., 2012; Marshall and Cahill, 2022). These intersecting barriers underscore the complex and multifaceted nature of healthcare disparities experienced by PWH, highlighting the urgent need for comprehensive interventions to address these systemic challenges and ensure equitable access to HIV care.

Furthermore, sexual and gender minorities, who are disproportionately affected by HIV (Jenkins et al., 2023; Clair et al., 2024; Scanlon et al., 2024), face distinct challenges, including societal stigma, gender-based violence and discrimination, and lack of culturally competent care, exacerbating barriers to HIV care and widening the health disparity gap among these marginalized communities (Wilson et al., 2013; Nelson et al., 2014). Addressing these multifaceted barriers is essential to ensuring equitable access to HIV care and improving outcomes in the HIV care continuum.

Structural interventions have proven to be effective in addressing social and structural conditions impacting HIV care (e.g., socio-ecological issues such as transportation and unemployment) (Powers et al., 2017; Miller et al., 2021; Johnson et al., 2022; Kapadia, 2022; Arreola et al., 2023; Coleman et al., 2023; Goldhammer et al., 2023), and a growing body of interventions seeks to address coping with discrimination and/or understand the impact of racism on health behaviors (Elligan and Utsey, 1999; Wilton et al., 2009; Bogart et al., 2018; Burns et al., 2024). An intervention that exemplifies this are Medical-Legal Partnerships.

Medical-Legal Partnership (MLP) is an effective approach to assist patients in addressing social and environmental issues that contribute to health disparities, with solutions found in civil law (Regenstein et al., 2018). MLPs have been successfully integrated into various health care settings, including federally qualified health centers, hospitals, VA medical centers, primary care clinics, and public health departments (Murphy, 2020). The implementation of MLPs has led to five significant outcomes: enhanced health outcomes and wellbeing, improved housing and utility stability, better access to financial resources, advancements in healthcare systems and workforce, and positive changes in policies, laws, and regulations (Murphy, 2020). MLPs bring together lawyers and health care professionals to address health-harming legal needs (HHLN), including discrimination, by providing improved linkages to legal support, which could lead to improvement in health outcomes and creates a more comprehensive and efficient system of care. A HHLN is a legal issue or challenge that negatively affects an individual's physical or mental health or their ability to access healthcare services (Berg et al., 2022; Johnson et al., 2024). These needs often intersect with social determinants of health and can include issues such as housing instability, discrimination, lack of health insurance, employment concerns, or access to disability benefits. Addressing health-harming legal needs is essential for promoting overall wellbeing and improving health outcomes (Gruskin et al., 2013; Atkins et al., 2014; Hall et al., 2022; Loughran, 2022; Girard et al., 2023; Liaw et al., 2023; Patchen et al., 2023).

MLPs in HIV care are an emerging approach that combines traditional medical care with legal support to address the HHLN of PWH (Martinez et al., 2017, 2022; Muñoz-Laboy et al., 2019; Yamanis et al., 2019). In other settings and with other populations including veterans, immigrants, formerly incarcerated individuals, children, and patients with cancer in palliative care (Weintraub et al., 2010; Sege et al., 2015; Tsai et al., 2017; Benfer et al., 2018; Selnau and Goldberg, 2019; Chen et al., 2021; League et al., 2021; Griesemer et al., 2023; Lu et al., 2023; Ramos et al., 2023), MLPs have been beneficial for addressing complex legal needs.

Building from this premise, this study aims to identify the challenges and benefits of MLPs in improving HIV care outcomes. Further, the study presents MLPs as an approach that addresses critical health-harming legal needs, including discrimination, among vulnerable populations, particularly sexual and gender minorities (SGM) living with HIV, with the ultimate goal of advancing equitable access to quality healthcare and reducing health disparities.

## Materials and methods

This exploratory study evaluates Medical-Legal Partnerships (MLPs) in HIV care, an area with limited research. Methods involved a secondary analysis of a cross-sectional survey administered to MLP providers serving people with HIV (PWH) in the United States, with a total sample size of 111 participants. Approval for the original research, the cross-sectional survey, was obtained from the Temple University Institutional Review Board (#25205).

### Recruitment

The recruitment process for the original research involved reaching out to a diverse range of professionals engaged in Medical-Legal Partnerships (MLPs), including administrators, clinicians, lawyers, and social/behavioral health service providers. Initially, the survey was distributed via email to providers associated with 294 MLP programs across the United States, all of which were registered with the National Center for Medical-Legal Partnership (NCMLP). Additionally, HIV administrators through U.S. Ryan White Care Clinics were identified and the survey invitation was extended to them as well. Emails included detailed information about the study's objectives and potential impact, emphasizing the importance of their participation in advancing understanding and improving HIV care outcomes. Administrators were encouraged to share the survey with their staff, thereby broadening the reach of our recruitment efforts. Providers were drawn from diverse professional backgrounds, encompassing fields such as behavioral health, medicine, administration, and law, and represented various types of organizations, including both for-profit and non-profit entities. To further enhance recruitment, social media networks were leveraged, including a dedicated MLP study account, to engage potential participants and disseminate information about the survey.

### Data analysis

Thematic analysis (Guest et al., 2012) was conducted on the responses gathered from open-ended short-answer questions focusing on the identification of challenges and benefits associated with MLPs. This method involved systematically identifying recurring themes and patterns within the responses to gain insights into the perceived advantages and obstacles of MLP involvement. The survey included questions such as “What are the challenges/disadvantages of being involved in an MLP?” and “What are the benefits/advantages of being involved in an MLP?” The analysis of responses was guided by the social ecological model (Williams et al., 2023), a theoretical framework that acknowledges the interconnectedness of various factors influencing health outcomes, including HIV care. This model facilitated a comprehensive examination of the multilevel determinants affecting the effectiveness of MLPs in HIV care settings. To streamline the analysis process, a coding scheme aligned with the social ecological model was developed (Baral et al., 2013; Centers for Disease Control and Prevention, 2015; Hergenrather et al., 2021), which provided a systematic approach to categorizing responses. Each response was independently coded into one or more of the five levels of the socio-ecological model: individual, interpersonal, organizational, community, and public policy factors.

Through this analysis, we aimed to provide a comprehensive understanding of the diverse experiences and perspectives of participants regarding MLPs and their impact on HIV care outcomes. Any ambiguities in coding were resolved through thorough discussion among the research team, comprising the first author and two co-authors, to ensure consensus in the coding process. This approach allowed for a nuanced exploration of the challenges and benefits of MLP involvement across various levels of influence, yielding valuable insights into the complex dynamics at play within HIV care.

## Results

Out of the 122 participating survey respondents, 11 were excluded due to appearing to be duplicates (10) or completely missing data (1). This brought the final sample size to 111. Providers included administrators (*n* = 17), clinicians (*n* = 25), lawyers (*n* = 40), and social service providers (*n* = 29). Some providers were from the same organization. 62.2% of providers were part of organizations identified as non-profit, 0.9% of providers were part of organizations identified as for-profit, and 36.9% of providers were part of organizations categorized as unknown (e.g., due to missing data on organization name, no apparent information regarding profit status online). Non-profit status was determined based on either the organization's IRS tax-exempt status (56.8% of providers) or other online sources (5.4% of providers). A summary of the final sample is shown in Table 1.

**Table 1:** Demographics.

**Variable**	**Count**	**Percentage**
**Provider's organization type**		
For-profit	1	0.9 %
Non-profit	69	62.2%
Unknown	41	36.9%
**Provider type**		
Administrators	17	15.3%
Clinicians	25	22.5%
Lawyers	40	36.0%
Social services providers	29	26.1%

### Benefits of MLPs: patient impact and benefits, comprehensive service provision, potential for policy influence, and enhanced staff competence

#### Patient impact and benefits

Several respondents highlighted significant benefits experienced by clients as a result of their engagement with Medical-Legal Partnerships (MLPs), particularly in terms of improving overall health outcomes and addressing individual needs. One notable benefit cited by participants was the positive impact on patient health, with one remarking, “…[increased] adherence” and another stating “identification of legal issues before they reach a crisis point and cause health crises.” These quotes underscore the preventive role of MLPs in managing health conditions and identifying and addressing legal issues proactively, thereby mitigating potential health-related complications for clients. Additionally, a provider emphasized the support provided by MLPs in mitigating stressors and preventing exacerbation of medical conditions, stating that “the MLP is invaluable in providing support to ensure that other issues do not cause stress or exacerbate the medical condition(s).” This observation highlights the holistic approach adopted by MLPs in addressing both legal and medical needs to promote overall wellbeing among clients. Moreover, participants noted various benefits related to fulfilling individual needs and enhancing financial stability. One participant highlighted the preventive nature of MLP interventions, stating, “we are able to prevent stress, displacement, loss of benefit or housing.” This quote underscores the role of MLPs in averting potential crises and safeguarding clients against adverse outcomes such as housing instability or loss of benefits. Additionally, MLPs were recognized for their role in reducing financial burdens associated with healthcare, with one participant noting that they “...reduce medical expense for someone already ill.” This sentiment underscores the economic benefits derived from MLP services, which not only improve access to healthcare but also alleviate financial strain for clients already grappling with health-related challenges. Overall, these quotes illustrate the multifaceted benefits of MLPs in addressing the diverse needs of clients and promoting their overall wellbeing and financial stability.

#### Comprehensive service provision

MLPs emerged as the preferred model for addressing health-harming legal needs and providing comprehensive services tailored to the needs of vulnerable populations. Participants highlighted the instrumental role of MLPs in expanding access to legal resources and the justice system for individuals living with HIV who are low-income. As one participant aptly stated, “The MLP provides low-income people living with HIV increased access to legal information and the legal system.” Another participant emphasized the convenience and accessibility afforded by MLPs as benefits, describing them as a “one-stop shop for patients who need services,” thereby addressing the needs of, as further described by the participant, individuals who may not “actively seek out legal aid” but have the “the convenience of the MLP.” Furthermore, participants underscored the significance of MLPs in facilitating access to competent social services, thus enhancing the overall quality of care provided to communities affected by intersecting forms of oppression. One participant highlighted the importance of MLPs in offering competent and sensitive care, stating that they contribute “...to our patients in being able to access competent social services....” This sentiment underscores the critical role of MLPs in addressing the multifaceted needs of marginalized communities and ensuring the provision of culturally competent care. Moreover, participants noted the positive impact of MLPs on improving the quality of life for patients, with one noting that they were “...quite helpful to learn more about patients [‘] quality of life….” Additionally, several participants expressed satisfaction with the comprehensive nature of services offered by MLPs, sharing a “comprehensive/great service” sentiment toward MLPs. This sentiment was echoed by a participant who emphasized MLPs' important trait of providing “more full service, whole person treatment,” highlighting the holistic approach adopted by MLPs in addressing the diverse needs of individuals. Overall, these findings underscore the pivotal role of MLPs in enhancing access to legal and social services, improving quality of life, and delivering comprehensive care to vulnerable populations, thereby addressing the complex challenges faced by individuals affected by health-harming legal needs and systemic inequalities.

#### Enhanced staff competence

The results revealed that MLPs significantly contribute to increasing staff competence and capacity to provide comprehensive care to patients. Many respondents highlighted the invaluable benefit of MLPs in equipping providers with enhanced knowledge, skills, and advocacy capacity to effectively address the diverse needs of patients. Specifically, participants noted that MLPs empower staff members to be well-informed in their work, which demonstrates MLPs ability to enhance providers' capacity to deliver high-quality services. One participant highlighted the remarkable advantage of staying “abreast of new knowledge and information” which is crucial for high-quality patient care. Another participant eloquently stated, MLPs provide “increased staff knowledge, skill, and advocacy capacity,” underscoring the transformative impact of these partnerships on staff development and professional growth. Furthermore, respondents emphasized the profound effect of MLPs in fostering a culture of continuous learning and knowledge acquisition across different types of providers. Participants expressed appreciation for the wealth of knowledge gained through their involvement in MLPs, highlighting the instrumental role of these partnerships in broadening their understanding of patients' legal needs and social determinants of health. One participant succinctly captured this sentiment by acknowledging that MLPs provide them with “a wealth of knowledge,” indicating the invaluable learning opportunities afforded by these collaborative initiatives. Moreover, MLPs were recognized for their role in early identification of patients' legal needs, thereby emphasizing MLPs critical role in facilitating timely intervention and support. A lawyer participating in the study emphasized the importance of MLPs in enabling providers to identify patients' legal needs at an early stage, underscoring the proactive nature of these partnerships in addressing potential challenges before they escalate. This proactive approach not only enhances patient care but also contributes to the overall effectiveness and efficiency of healthcare delivery within the MLP framework. Overall, these findings underscore the transformative impact of MLPs in enhancing staff competence, knowledge, and advocacy skills, ultimately leading to improved patient care and outcomes. By equipping providers with the necessary tools and resources to address patients' legal needs proactively, MLPs play a vital role in promoting holistic and patient-centered care within healthcare settings.

#### Potential for policy influence

Though not a significant theme, the appearance of public policy concerns suggests that the impact of MLPs on public policy should be further explored, as they have a unique role in seeking solutions at both the individual and policy levels. As one participant succinctly stated, “they seek out solutions at the individual and policy levels,” indicating the dual focus of MLPs on addressing immediate legal needs while also advocating for broader systemic change. While this statement did not provide specific details, it underscores the proactive approach adopted by MLPs in addressing legal barriers to healthcare access and advocating for policy reforms to improve health outcomes for vulnerable populations. Furthermore, the unique ability of MLPs to influence public policy and disrupt the cycle of individuals returning to unhealthy conditions that perpetuate their reliance on clinical or hospital care was highlighted by the same participant. They aptly described MLPs as being “...uniquely qualified to help the healthcare system disrupt the cycle of returning people to the unhealthy conditions that would otherwise bring them right back to the clinic or hospital.” This statement emphasizes the role of MLPs in addressing the root causes of health disparities and promoting sustainable solutions that address social determinants of health. Overall, these limited findings related to public policy call for the need to focus future research on the important role of MLPs in driving policy change and advocating for systemic reforms that address the underlying social and structural factors contributing to health inequities. By engaging in policy advocacy and promoting innovative solutions, MLPs contribute to the broader goal of creating a more equitable and inclusive healthcare system that meets the needs of all individuals, particularly those from marginalized and underserved communities.

### Challenges of MLPs: funding and healthcare systems limitations, resource integration and limitation issues, and staffing issues and constraints

#### Funding and healthcare systems limitations

Funding emerged as a significant hurdle to the successful implementation and long-term viability of Medical-Legal Partnerships (MLPs). One participant lamented the “lack of sustainable funding,” while another underscored the issue by stating, “there is limited funding…” Another participant succinctly emphasized the critical nature of funding by reiterating, “FUNDING. FUNDING. FUNDING.” Participants responses conveyed the importance of securing both local and federal funding to ensure the sustainability of MLPs over time. Regarding the challenges posed by the existing U.S. healthcare system and organizations within it, one participant highlighted, “some challenges include the health care system to which we are under…” underscoring the complexities inherent in navigating the current healthcare landscape. Similarly, another participant noted that “working with medical systems can be challenging,” shedding light on the difficulties encountered when collaborating within the framework of the healthcare system. However, the participants' responses regarding funding and healthcare system challenges were limited, leaving gaps in information such as which funding streams are most challenging and the specific types of challenges posed by the system and at what level. Therefore, these areas should be further explored to ensure the sustainability of MLPs.

#### Resource integration and limitation issues

Resource integration and limitations appeared as significant challenges facing Medical-Legal Partnerships (MLPs). Participants highlighted the need for improved accessibility and integration of legal services within healthcare settings. One participant expressed frustration, stating, “I wish we had a [ORGANIZATION] lawyer on site here as well! Sometimes it can be tough to reach them over the phone,” underscoring the logistical hurdles encountered in accessing legal support. Another participant emphasized the scarcity of resources, lamenting that “there is unfortunately not enough resources to go around for individuals that are mostly in need.” Lawyers, in particular, highlighted the constraints imposed by limited resources and the fragmented nature of services. One participant commented on the challenges posed by the “lack of full integration in medical team” and explained how this “creates communication and training barriers.” Additionally, a participant noted that “access to healthcare professionals is limited, indicating an unstable MLP integration,” demonstrating the challenge of incorporating MLPs within the healthcare system. These quotes illuminate the difficulties faced in effectively integrating legal services into healthcare settings, pointing to the need for enhanced resource allocation and seamless collaboration between legal and medical professionals within MLPs.

#### Staffing issues and constraints

Staffing issues and constraints emerged as significant challenges within MLPs, with participants highlighting various obstacles related to limited staffing resources and operational capacity. One participant underscored the staffing limitations, stating, “we have only been operating for 2 years, and we are a staff of two, one MLP attorney and one program coordinator. We could already use another attorney.” This quote illustrates the strain imposed by insufficient staffing levels, hindering the MLP's ability to meet the diverse legal needs of clients effectively. Another participant emphasized the disparity between legal caseloads and available resources, noting, “from the legal perspective, one of the biggest challenges is meeting all of the legal need that presents at the MLP. Legal cases take time and one lawyer cannot handle as many cases a one doctor sees patients.” This observation underscores the mismatch between the volume of legal issues requiring attention and the limited capacity of MLPs to address them adequately. Additionally, staffing constraints were exacerbated by limited operational hours, as highlighted by a participant who remarked, “we are only there 2 out of 5 days, so if a patient does not seek referral through a provider and stop-in, they might not get assistance and never come back if we physically were not present.” This quote emphasizes the critical importance of consistent availability in providing legal assistance to clients, particularly those who may face challenges in accessing services during limited operating hours. Furthermore, one participant noted, “...low pay and high student loans,” highlighting the financial constraints and pressures experienced by MLP employees. Lastly, communication breakdowns between legal and medical teams were cited as a challenge, with one participant expressing frustration: “Medical providers' policies aren't always clear, and instead of being straightforward about whether or not they can help us, we get sent back and forth between a number of supervisors and administrators to the point where it affects our representation.” This quote underscores the importance of streamlined communication processes to facilitate collaboration between legal and medical professionals within MLPs and ensure efficient service delivery to clients.

## Discussion

To our knowledge, this study represents the first comprehensive exploration of the challenges and benefits encountered by MLPs serving the needs of PWH. The insights gleaned from this research have far-reaching implications for clinical practice and the broader endeavor of advancing health equity within vulnerable communities. Specifically, these findings demonstrate that MLPs are a promising approach to addressing the diverse needs of PWH, including those arising from structural stigma and other systemic inequities. Sexual and gender minorities, who often face the compounded challenges of HIV, stigma, discrimination, housing instability, and lack of access to comprehensive care (Flentje et al., 2017; Putney et al., 2021; Gleason et al., 2023; Wiginton et al., 2023; Fisk-Hoffman et al., 2024), can particularly benefit from the MLP approach. This approach focuses on addressing upstream barriers to care through comprehensive legal interventions, making it a critical tool in the effort to improve health outcomes for underserved populations.

The findings highlight several organizational-level challenges faced by MLPs, including funding constraints, healthcare system limitations, resource integration issues, and staffing constraints. Funding limitations pose a significant barrier to the sustainability and effectiveness of MLPs (Rubin, 2019; Gallen et al., 2023; Kraschel et al., 2023; Johnson et al., 2024), hindering their ability to address the complex legal needs of vulnerable populations, including SGM individuals living with HIV. Without adequate funding, MLPs may struggle to provide essential legal services and advocacy, exacerbating disparities in access to healthcare and justice. Moreover, the challenges posed by the existing healthcare system, such as bureaucratic hurdles and limited resources, further compound the difficulties faced by MLPs in meeting the needs of people with HIV. These systemic barriers underscore the need for increased investment in MLPs and reforms to the healthcare system to ensure equitable access to legal and healthcare services.

To ensure the long-term sustainability of Medical-Legal Partnerships (MLPs), it is crucial to explore various strategies that leverage existing resources and foster new collaborations. One effective approach is to establish partnerships with law school clinics, which typically have summer interns and access to pro-bono attorneys. Many law school clinics also have networks of alumni and practicing attorneys willing to offer pro-bono services. Engaging these attorneys can provide MLPs with additional legal expertise without associated costs. In addition to law school clinics, expanding pro-bono networks can further support MLP sustainability. Strategies include partnering with local bar associations to create a roster of attorneys willing to offer pro-bono services to MLP clients. These collaborations can provide a steady stream of legal expertise and support for MLPs, enhancing their capacity to address the legal needs of patients.

Resource integration issues within MLPs present additional challenges, particularly concerning the accessibility and coordination of legal services within healthcare settings. PWH may encounter difficulties in accessing legal support due to fragmented services and communication barriers between legal and medical professionals. This lack of integration can impede timely intervention and exacerbate health disparities among marginalized populations. Addressing these resource integration challenges is essential to improving the accessibility and effectiveness of MLPs for PWH, ensuring they receive comprehensive and coordinated care that addresses their unique legal and healthcare needs.

Staffing constraints within MLPs further hinder their ability to serve vulnerable populations effectively. Limited staffing resources and operational capacity may result in delays in accessing legal assistance, particularly for SGM individuals living with HIV who may face multiple intersecting legal challenges. Additionally, inadequate staffing levels can compromise the quality of care provided by MLPs, impacting the overall wellbeing of clients. To address these challenges, MLPs must prioritize recruitment and retention of diverse staff members with varying availabilities, including those with expertise in serving SGM populations, to ensure culturally competent and responsive care.

Findings also highlighted the significant benefits of MLPs for PWH, underscoring their positive impact on patient health outcomes, including the early identification of legal issues that may affect health. Early detection of legal needs is beneficial for several reasons. For instance, preventing an eviction can prevent homelessness. Additionally, preventive legal aid through educational “know your rights” workshops can help patients identify early legal needs or take proactive legal action to address ongoing harms they might not have considered needing legal intervention or thought had legal remedies. For example, a transgender woman experiencing discrimination in the workforce might not be aware of local, state, or federal protections she could seek to prevent employment discrimination. By attending a “know your rights” workshop, she might learn about these protections and be linked to an attorney to intervene. By addressing the social determinants of health, MLPs can mitigate the adverse effects of discrimination and marginalization experienced by PWH with multiple intersecting identities, thereby promoting their overall wellbeing and resilience.

Furthermore, MLPs provide comprehensive services that address the intersecting needs of vulnerable populations, including legal, medical, and social support. This holistic approach ensures that PWH receive integrated care that addresses their unique circumstances and promotes empowerment. MLPs serve as a vital resource for PWH facing discrimination and legal barriers to healthcare access, offering advocacy, education, and support to navigate complex legal systems and assert their rights.

Additionally, MLPs have the potential to influence public policy and advocate for systemic reforms that address discrimination and promote health equity (Zisser and Stone, 2015; Shah, 2024). By engaging in policy advocacy and community organizing, MLPs can amplify the voices of marginalized populations, including PWH, and advocate for policies that advance social justice and equality. One notable example of the transformative impact of MLPs on healthcare and legal practice is the Health Justice Project in Chicago, Illinois (https://www.luc.edu/law/academics/clinical-programs/healthjusticeproject/). This project has been recognized for its innovative approaches and significant contributions to the field. Among its key innovations is a logic model study that evaluates the effect of MLPs on achieving health equity. This study has provided valuable data supporting the integration of legal services into healthcare to improve health outcomes for marginalized populations. Additionally, the Health Justice Project was invited by the Illinois Supreme Court Access to Justice Commission to draft legislation aimed at improving access to justice for underserved communities. This invitation highlights the project's expertise and the critical role it plays in shaping policies that promote health equity and protect the rights of vulnerable populations. Furthermore, the Transgender Legal Defense & Education Fund (TLDEF) is a prominent organization that works to achieve equality for transgender people through impact litigation, direct legal services, public policy advocacy, and education (https://transgenderlegal.org/about-tldef/). One of their notable initiatives is TLDEF's Impact Litigation Program, which brings cases and files amicus briefs (friend-of-the-court briefs) in state and federal courts across the country to protect the rights of transgender and non-binary people. They take on cases that offer courts opportunities to establish new precedents. TLDEF's impact litigation work focuses on defending and uplifting transgender and non-binary individuals who have limited access to legal services and who face intersecting forms of discrimination, including due to race and ethnicity, socioeconomic class, geography, and lack of access to benefits and services. Through strategic partnerships and coalition-building, MLPs like the Health Justice Project and TLDEF can leverage their expertise to inform legislative and regulatory initiatives. By collaborating with healthcare providers, legal professionals, community organizations, and policymakers, MLPs create a unified front against social injustices that affect health. These collaborations are essential for developing and implementing policies that address discrimination and ensure access to healthcare.

### Limitations

This study has several limitations. First, the study's cross-sectional design limits the ability to establish causality or assess long-term outcomes of MLPs. Future research employing longitudinal designs could provide valuable insights into the sustained impact of MLPs on health outcomes and addressing structural racism and discrimination. Second, the study's reliance on self-reported data may introduce response bias or social desirability bias, potentially influencing the accuracy of participants' responses. Third, the generalizability of the findings may be limited by the study's focus on MLPs serving individuals living with HIV in the U.S. MLPs operate within diverse healthcare contexts globally, and so, experiences and challenges may vary across different settings and populations. Future research should explore the applicability of these findings to other contexts and populations, including SGM individuals living with HIV in low- and middle-income countries, to inform culturally responsive and contextually appropriate interventions.

## Conclusion

Study findings highlight the indispensable role of MLPs in combatting discrimination and advancing health equity among PWH, particularly those navigating multiple intersecting identities, including sexual and gender minorities. By addressing organizational-level challenges and leveraging their unique strengths, MLPs can continue to serve as vital resources for vulnerable populations, offering comprehensive care, advocacy, and support to address the complex needs of individuals facing discrimination and marginalization. Through strategic partnerships, policy advocacy, and community engagement, MLPs can contribute to broader efforts to advance social justice, equality, and inclusion, creating a more equitable healthcare system.

## Data availability statement

The datasets presented in this article are not readily available given the sensitive nature of the data, including HIV data, we would not like to make the dataset public. Requests to access the datasets should be directed to OM, omar.martinez@ucf.edu.

## Ethics statement

The studies involving humans were approved by Temple University Institutional Review Board. The studies were conducted in accordance with the local legislation and institutional requirements. The participants provided their written informed consent to participate in this study.

## Author contributions

JJ: Conceptualization, Data curation, Formal analysis, Investigation, Methodology, Project administration, Writing – original draft, Writing – review & editing. AFra: Supervision, Writing – review & editing. AFre: Writing – review & editing. RD: Writing – review & editing. MM-L: Writing – review & editing. OM: Supervision, Writing – original draft, Writing – review & editing.
